# Instinct versus Discipline

**Published:** 1900-04-14

**Authors:** 


### Instinct versus Discipline.

Among the attributes which man shares with the brutes is instinct, and among the instincts which civilised man shares with savages is that of self-preservation in the face of danger. But just as man moves far less than is the case with the lower animals in grooves of instinct, so does civilised man, living far removed from danger, possess in much less degree than his more savage brethren those instincts of caution which make for safety in times of war. Savage man is everywhere distinguished by the caution with which he approaches the unknown, and the extraordinary sharpness with which he observes the slightest indication of the presence of an enemy. Fitness to survive is not with him merely a matter of strength and courage, but of those attributes of cunning, acuteness, and readiness to fight when brought to bay which we may group together as instincts of self-preservation. Sometimes, measuring him by other standards of honour, we are apt to think that the savage is a poor creature, so marked is his tendency to avoid danger rather than to face it, and to sneak and lie in ambush rather than engage in open battles. These, however, are the instincts which long experience has stamped upon his brain, and by virtue of which he has survived. Civilisation alters all this, and not always for the better. By long centuries of the reign of law, and as a consequence of the trustfulness which is born of inexperience of danger, many of our instincts of self-preservation have become dulled; and our soldiers being recruited from among peaceful citizens, discipline has had to take the place of discarded instincts. War having become a matter of intellect, discipline, which is the very abnegation of instinct, has become essential; but between opponents with equal arms it may be doubted whether the most perfect and machine-like obedience to the single will of the commander is a full substitute for the co-operation of many minds all acting in response to combative and self-protecting instincts, which have, during long years of trial, become engrained upon them as a racial endowment. Of this we have had abundant experience during the war in South Africa.

Of the valour of our race, of the hardihood of our troops, and of their splendid gallantry in doing and daring everything that can be put before them we have had unending proof. England can indeed, if she so pleases, plume herself upon the boundless bravery of her sons, even while mourning the death of so many of the best among them. But if war is to be regarded as a means to an end, and if each act in the conduct of a war is to be measured by the extent to which it conduces to that end, it is clear that much that has happened in South Africa, and much, be it added, which has appealed most strongly to the feelings of a nation which always places courage on the highest pedestal of honour, has been absolutely thrown away. When, in the name of discipline and duty, one finds men standing up to be shot at by an unseen foe; when men are seen rushing time after time against trenches which it is impossible to reach through the withering fire pouring out on every side; and when, in open disregard of possible dangers, we find whole companies walking, under the guidance of their officers, into traps deliberately laid for them, we must have our doubts whether the discipline which leads to such heroic but useless loss is a full substitute for that instinct which teaches our enemies to be cautious before all things, to dig, and trench, and lie in wait, and even run away—things to which we give hard words, but which, for all that, make them difficult to overcome.

We do not wish to call the Boers savages. The last thing in the world which we would do would be to join in the chorus of those who see nothing but evil in the enemy. But the fact remains that for generations the life of the Boers has been such as to develop in the highest degree those instincts of self-preservation which are natural to savage man, even the instinct of duplicity. Meanwhile in England men have slept in peace; window shutters have gone out of fashion, and the instinct of self-preservation in the face of suspected danger has atrophied from want of use. Can we then expect discipline to meet instinct upon an equal footing? Can we expect a good-natured soldiery, who so far as inborn instinct is concerned would rather give a dusty Boer a glass of ale than shoot him, to contend on equal terms, by dint of discipline, with a people who have been forced by hard necessity to keep in full activity the wily self-protective instincts of the savage? As a mere problem in sociology we should say "No." The problem is an old one. Other empires have faded, and it has been held that they have faded from effeminacy. Perhaps it would be more true to say that they have collapsed from their fighting men losing the fighting instincts which more than mere discipline bring success. This instinct is always best developed along the fringe of empire—now far removed beyond the borders of the mother country—and it may be that England's greatest chance of permanency lies in such consolidation of the empire that her soldiers shall be largely recruited from the outer zone, but shall enter her army not as mercenaries but as brothers.

